# Emotion-focused dyadic coping styles used by family carers of people with dementia during the COVID-19 pandemic

**DOI:** 10.1177/14713012231173812

**Published:** 2023-05-05

**Authors:** Carmen Colclough, Eleanor Miles, Jennifer Rusted, Rotem Perach, Ben Hicks, Josie Dixon, Margaret Dangoor, Kate Gridley, Yvonne Birks, Paul Donaghy, Riona Mcardle, Elen Moseley, Harsharon K Sondh, Sube Banerjee, for the DETERMIND team

**Affiliations:** School of Psychology, 1948 University of Sussex, Brighton, UK; School of Social Sciences, 4921 University of Westminster, London, UK; 12190 Brighton and Sussex Medical School, Brighton, UK; 1948 University of Sussex, Brighton, UK; Care Policy and Evaluation Centre, 192363 London School of Economics and Political Science, London, UK; Social Policy Research Unit, 8748 University of York, York, UK; Translational and Clinical Research Institute, Faculty of Medical Sciences, 5994 Newcastle University, Newcastle upon Tyne, UK; 4958 South London and Maudsley NHS Foundation Trust, London, UK; Faculty of Health, 6633 University of Plymouth, Plymouth, UK

**Keywords:** dementia, carer, dyadic coping, qualitative, COVID-19

## Abstract

Emotional wellbeing of family carers and people with dementia is associated with not only how each individual copes with stress and conflict, but also by how they cope together. Finding ways to positively cope together was particularly important during COVID-19 lockdown restrictions, when other avenues of emotional support were less available. We explored how carers experienced and used emotion-focused dyadic coping styles during the COVID-19 pandemic. In-depth qualitative interviews were conducted during the pandemic with 42 family carers, supplemented by quality of life scores collected both pre- and during the pandemic and household status. Abductive thematic analysis identified five styles of emotion-focused dyadic coping: common, supportive, hostile, disengaged avoidance and protective. The COVID-19 pandemic left many dyads unsupported. While many carers adapted, reporting increases in quality of life and enjoying the extra time with the person with dementia, others experienced dyadic conflict and reductions in quality of life. This variation was associated with dyadic coping styles, including challenges in using ‘positive’ styles and the protective use of ‘negative’ disengaged avoidance in the right situations. Dyadic coping styles also differed as a function of whether the dyad lived together. As many people with dementia are supported by an informal carer, considering how they cope together could help us to better support them. We make suggestions for dyadic interventions tailored by co-residency status that could help dyads identify and communicate coping needs, reconnect following avoidance coping, and replenish their coping resources through social support.

## Introduction

Many people with dementia and their carers experienced negative emotions and reduced quality of life during the COVID-19 pandemic (Hicks et al., 2022; Losada et al., 2021). Conversely, some of these dyads adapted well (Hanna et al., 2021) with some even reporting an increase in positive emotions (Losada et al., 2021). Success in adapting to lockdowns has been partly attributed to practical coping strategies, such as walking in green spaces or maintaining a routine (Bacsu et al., 2021). Given that a substantial majority of people with dementia are supported by an informal carer (Lewis et al., 2014) and that emotional distress is common in dementia dyads (Kuring et al., 2018; Svendsboe et al., 2018), understanding dyadic coping processes could help explain variation in how dyads successfully adapted to lockdowns. Dyadic coping styles, as presented in Falconier and Kuhn’s integration model (2019), have been associated with relational and quality of life outcomes for dyads coping with long term illness (Vaske et al., 2015; Zimmermann et al., 2010). However, little is known about how these styles are used and experienced in the context of dementia. Understanding these coping processes could provide much needed insight into effective dyadic-focused interventions to promote adaptive coping. This could provide dementia dyads with skills to enable them to maintain their quality of life, emotional wellbeing, and the dyadic relationship at home. We interviewed family carers to explore these coping dynamics, considering challenges and facilitators for positive coping when one person has dementia, and when the dyad was isolated due to COVID-19 lockdown restrictions.

### Emotion-focused dyadic coping

The dyadic coping approach views coping as a relational and transactional process, in which dyads signal stress to each other and react with a coping response to reduce stress relevant to the dyad (Bodenmann et al., 2011). Understanding dyadic coping processes provides insights into how people in close relationships regulate emotional states together to protect the wellbeing and relationship quality of the dyad. Falconier and Kuhn’s integration model (2019) synthesises two decades of research on dyadic coping. Each style refers to an approach to dyadic coping (Table 1), for example dyads may approach stress together (common), one may provide the other with support (supportive), or dyads may avoid each other in times of stress (disengaged avoidance). This model groups coping styles used by the dyadic unit as ‘positive’ or ‘negative’, dependent on how adaptive they are deemed to be. A range of specific coping strategies can be used within each of these styles (Carver et al., 1989; Lazarus & Folkman, 1987). For example, a hostile (‘negative’) coping style may include strategies such as blame or expressions of anger, while listening or perspective taking could be used within a supportive (‘positive’) coping style. Emotion-focused coping involves an attempt to change the meaning of a stressor through methods such as reframing, avoidance or humour to modify affective state (Lazarus & Folkman, 1987). Given that emotional distress is common in people with dementia (Kuring et al., 2018) and their carers (Svendsboe et al., 2018), developing strategies to regulate the emotional state of the dyad is important for both members’ wellbeing. Identifying the dyadic coping strategies used by carers could help us understand dyadic coping processes and inform interventions which could promote adaptive coping.

**Table 1.** Emotion-focused dyadic coping styles as described by Falconier and Kuhn’s (2019) integration model.

Coping Styles	Brief definition
Positive	
**Active Engagement**	Conversations with partner about how they are feeling
**Supportive** (Empathic responding)	One partner providing support to the other
**Common** (Collaborative, communal, Mutual Responsiveness)	Actively dealing with stress together
Negative	
**Overprotection**	Shielding partner from stressors
**Protective Buffering**	Concealing one’s own emotions to protect partner
**Hostile** (Ambivalent)	Unsympathetic responses or insincere support
**Disengaged Avoidance** (Common Negative)	Avoiding dyadic stress or each other

*Note.* The first term (bolded) for each coping style is the term we use in this paper. Additional terms (in brackets) are coping styles identified in the literature by Falconier and Kuhn (2019) that conceptually overlap. From the original model, we have omitted delegated and controlling dyadic coping styles as they can be considered problem-focused. Adapted from *“Dyadic Coping in Couples: A Conceptual Integration and a Review of the Empirical Literature”* by M. Falconier and R. Kuhn, 2019, Frontiers in Psychology, 10, 571, p. 8. Copyright 2019 by Frontiers.

### Emotion-focused dyadic coping in dementia

There is a growing call to consider dementia dyads as a unit in order to better understand how these interdependent relationships function and how dyads cope with the challenges of dementia together (Orsulic-Jeras et al., 2020). Currently we know very little about how dyadic coping is used and experienced when one person has dementia. Given the progressive nature of most dementias, often including changes in emotional experiences and expression (Henry et al., 2009), dementia dyads may need to continually adapt their coping efforts to help support each other through the changes. Kristofferzon and colleagues (2018) argues that emotion-focused coping can be particularly beneficial for situations that cannot be controlled, such as the COVID-19 pandemic and progression of dementia. Currently, research on dyadic coping in dementia during the COVID-19 pandemic has mainly focused on practical coping, such as going for walks or maintaining a routine (Bacsu et al., 2021), while we know little about how dyads emotionally coped together during this period. Understanding how dementia dyads use emotion-focused coping to deal with stressors together could help identify modifiable factors in dyadic coping that support positive adjustment and relationship maintenance.

### Dyadic coping challenges and facilitators

Positive dyadic coping is associated with increased wellbeing and relationship satisfaction for healthy dyads (Falconier & Kuhn, 2019). However, associations between ‘positive’ dyadic coping and positive outcomes appear less straight forward for dyads coping with long-term illness. For example, when female carers in cancer dyads used common coping to deal with stressors they shared with their partner, increases in negative affect in the partner were also experienced by the carer (Berg et al., 2011). Likewise, Rottmann and colleagues (2015) found that when partners of cancer patients reported receiving supportive dyadic coping, depression was higher 5 months later. The authors suggest this could be because caregiver partners may feel that it should be the patient receiving support, not them. In dementia, emerging quantitative evidence has found that positive dyadic coping styles are associated with burden and depressive symptoms for the person with dementia and their carer, but findings so far are mixed and limited (Connelly et al., 2020; Gellert et al., 2018). For example, contrary to expectations, Gellert and colleagues (2018) found that supportive coping by carers was associated with depression in the person with dementia. The authors suggest the person with dementia may receive supportive coping as unwanted or perceive it as threatening their autonomy. Dyadic coping in the context of dementia is likely to expose unique processes. Symptoms associated with dementia may change how the dyad has historically coped together. For example, carers report that mutual understanding becomes challenging due to reduced communication and insight associated with the progression of dementia (O’Shaughnessy et al., 2010). Additionally, some ‘negative’ avoidance strategies that involve humouring or agreeing can be used to validate the person with dementia’s experience, thus reducing agitation (Scales et al., 2018). More research is needed to determine how situational, relational, and emotional factors shape and guide the use of dyadic coping for dementia dyads, and their different outcomes. Mapping these processes could inform interventions to support dementia dyads as well as help professionals to identify and support dyads struggling to cope together.

### The current study

We conducted in-depth qualitative interviews with family carers of people with dementia during the COVID-19 pandemic about their experiences, coping, and relationship during this period. One of the aims of these interviews was to identify and understand the emotion-focused dyadic coping styles used by family carers, and to consider how this knowledge could provide carers and people with dementia the skills to cope together. This paper presents the findings of the analysis pertaining to this aim.

## Methods

### Participants

Carers were recruited from the DETERMIND C-19 cohort, a study exploring how quality of life and wellbeing of people with dementia and their carers were impacted by the COVID-19 pandemic. The DETERMIND C-19 study was nested within DETERMIND (DETERMinants of quality of life, care and costs, and consequences of INequalities in people with Dementia and their carers; Farina et al., 2020) programme of research which recruited participants from three areas of England (Sussex, North East, South London). From 114 family carers participating in the DETERMIND C-19 study in 2020, a purposive sample of 68 carers were contacted for maximum variation, ensuring a range of participants with varying quality of life scores (C-DEMQoL; Brown et al., 2019) and demographics (age, ethnicity, gender, relative socio-economic deprivation, and educational background). From this, 42 carers agreed to take part in the interviews (see Table 2).

**Table 2.** Sampling table of carer characteristics.

Characteristics	Co-resident (*N* = 20)	Non-co-resident (*N* = 22)
	n	n
Age		
20–34	1	1
35–49	1	2
50–64	2	11
65–79	9	8
80 and above	7	0
Gender		
Female	14	18
Male	6	4
Relationship to person with dementia		
Spouse	19	0
Daughter	0	15
Son	0	4
Granddaughter	0	2
Grandson	1	0
Sister	0	1
Area		
Urban	15	18
Rural	5	3
Missing data	0	1
C-DEMQoL from baseline to follow up^a^		
Maintained	2	2
Decreased	11	7
Increased	6	11
Missing data	1	2
Type of dementia		
Alzheimer’s disease	9	16
Vascular dementia	4	2
Mixed	3	2
Other/Unknown	4	2
Employment Status in pandemic		
Full-time carer	1	3
Employed	0	3
Self-employed	1	0
Volunteer	1	1
Retired	13	7
Unemployed	1	5
Made redundant	2	1
Furloughed/reduced working hours	1	1
Change in employment	0	1
Accommodation status		
Homeowner	17	15
Council rented	1	3
Housing association rented	0	3
Other	2	1
Index of multiple deprivation quintile		
1 (most deprived)	2	5
2	2	3
3	7	4
4	5	3
5 (most affluent)	4	5
Missing data	0	2
Ethnicity		
Asian (unspecified)	0	1
Caribbean	0	1
Indian	1	0
White/Black African	1	0
White/Black Caribbean	0	1
White British	18	19

^a^Within 1 point on the C-DEMQoL scale (see Table 3 for individual trajectories).

### Procedure and data collection

In-depth interviews were conducted with carers either by phone or video call (following COVID-19 safety protocol). Interviews took place between November 2020 and January 2021, while lockdown restrictions and stay-at-home measures were in place in the UK. Participants were sent consent forms and information about the study prior to interview and were given at least a week to consider their participation. A member of the research team then rang them to ascertain willingness to participate in the study and obtain verbal consent, which was recorded electronically. A team of three experienced researchers led the interviews (BH, JD, KG) guided by a topic guide developed collaboratively by the research team (see Supplementary Appendix 1). The topic guide begun with open questions and had prompts to stimulate in-depth discussion around the carer’s experiences during the COVID-19 pandemic, including topics relevant to dyadic coping (i.e., ways they were supporting the person with dementia and how the pandemic had impacted their relationship). Researchers conducted the interviews with flexibility, creating space for the participants to discuss experiences that were significant to them and then probing for more depth to ensure a fuller understanding (Rubin & Rubin, 2011). This allowed for a deep exploration of the topic and provided a participant-led representation of their lived experiences. Carer quality of life was measured using C-DEMQoL (Brown et al., 2019), a well validated instrument designed to measure quality of life specifically for those who care for a person with dementia. Total scores range from 30-150, with higher scores indicating better quality of life. C-DEMQoL data were also available from baseline assessment (pre-pandemic) and descriptive individual trajectories for each participant are presented in Table 3. Interviews ranged from 31-101 min long; participants were informed that they could take a break or withdraw at any time. Interviews were recorded and transcribed with identifying personal information removed or changed to protect anonymity of participants.

**Table 3.** Carers’ descriptive individual trajectories of change in quality of life scores (C-DEMQoL).

Non-co-residents (*N* = 22)			Co-residents (N = 20)		
Family carer	C-DEMQoL Baseline (follow-up)	C-DEMQoL change in score	Family carer	C-DEMQoL baseline (follow-up)	C-DEMQoL change in score
Daughter A	84 (97.5)	13.5	Wife A	103.45 (83.79)	−19.66
Daughter B	75 (89)	14	Wife B	105 (101.79)	−3.21
Daughter C	127.2 (130.34)	3.14	Wife C	113 (102.22)	−10.78
Daughter D	120 (115)	−5	Wife D	78.89 (78)	−0.89
Daughter E	83.48 (90)	6.52	Wife E	87.86 (83)	−4.86
Daughter F	115.56 (120)	4.44	Wife F	88.89 (87.78)	−1.11
Daughter G	74 (76)	2	Wife G	122.07 (134.44)	12.37
Daughter H	103.45 (103.85)	0.4	Wife H	88 (95)	7
Daughter I	NA (87)	NA	Wife I	90 (82)	−8
Daughter J	106 (113)	7	Wife J	85.86 (85)	−0.86
Daughter K	86 (113.57)	27.57	Wife K	94.14 (90)	−4.14
Daughter L	116 (115.86)	−0.14	Wife L	107 (80)	−27
Daughter M	NA (88)	NA	Wife M	139.2 (141.11)	1.91
Daughter N	92.07 (88.85)	−3.22	Wife N	96.21 (98.28)	2.07
Daughter O	115 (92)	−23	Husband A	NA	NA
Son A	105 (82)	−23	Husband B	100 (85)	−15
Son B	112 (124.29)	12.29	Husband C	104.44 (98.28)	−6.16
Son C	120 (110)	−10	Husband D	113.79 (116.67)	2.88
Son D	75 (76)	1	Husband E	91.11 (89)	−2.11
Sister A	114 (104.4)	−9.6	Grandson A	85 (88.97)	3.97
Granddaughter A	112.22 (106.8)	−5.42			
Granddaughter B	93 (130.34)	37.34			

*Note.* NA is missing data. Quality of life baseline scores were obtained prior to the Covid-19 pandemic (September 2019 - March 2020) and follow up scores were taken during Covid-19 at the time of the interview (November 2020 - January 2021).

Deidentified data from this study will be formally deposited with the UK Data Service after the last DETERMIND-C19 publication. This is expected to be around June 2023 and will be accessible at https://ukdataservice.ac.uk/find-data/.

Before June 2023, data will be available by contacting determind@bsms.ac.uk or by making a request through the project website at https://determind.org.uk/ providing the proposed use of the data has been approved by the DETERMIND Programme Management Board.

### Data analysis

The lead author employed an abductive thematic approach (Thompson, 2022) using a framework to coordinate data management across the team (Ritchie et al., 2013). The researchers who led on data collection (BH, JD, KG) developed the initial framework (with a row per participant and columns with general topic headings) based on initial familiarisation with the data, and refined through discussions with the DETERMIND team (13 researchers with varied demographics and research interests). A sample of transcripts were summarised into the chart and further refinements of the framework made before the remaining transcripts were allocated to the 13 researchers to chart between them. The framework, at this stage, included broad descriptive codes prepared to support a range of analyses pertaining to carers’ experiences during the pandemic. The initial framework did not include a specific code for dyadic coping, rather crosscutting themes relating to coping were developed subsequently during more focused analysis by the lead author. The entire framework was considered for analysis to ensure a comprehensive view of the data. This more focused analysis was conducted by the lead author, guided by Thompson’s stages of abductive thematic analysis. An overview of this process is detailed in Table 4.

**Table 4.** Thompson’s (2022) eight stages of abductive thematic analysis.

Stages	Actions
1. Familiarisation	The first author read the entire framework several times for familiarisation of the data. Due to the large quantity of data and range of topics discussed in interviews not relevant to this particular analysis, data unrelated to coping, the relationship, or the carers emotional state was removed from the framework for this analysis.
2. Coding	Examples of carer’s dyadic coping along with associated experiences and circumstantial factors were coded to capture how dyadic coping was utilised and experienced by carers. Coding at this stage was broad and inductive relating to anything carers discussed about how they were coping as a dyad.
3. Codebook	A list of codes was created and updated throughout the analysis (see Supplementary Appendix 2). Stage 2 was repeated to ensure everything was captured.
4. Development of Themes	Mind maps were created to cluster codes (strategies) into coping-related themes (styles). Themes were iteratively reviewed and discussed with members of the DETERMIND team (EM, RP, BH) until the final themes were agreed using the Falconier and Kuhn’s model (2019) as a guide.
5. Theorising	We began abductively shifting (Thompson, 2022) between our initial reading of the data and a more theoretically informed reading of the data applying Falconier and Kuhn’s model (2019). We examined the data coded for each theme to determine facilitators and challenges associated with each style of coping.
6. Comparison of Datasets	A cross case analysis was carried out to compare carers’ coping styles with their living situation (co-resident or non-co-resident), and changes in their C-DEMQoL scores from pre-pandemic to the time of interview. These data were examined at an individual level to characterise change in carer C-DEMQoL from baseline to follow-up interview, Table 3 presents these individual trajectories of change in C-DEMQoL scores for the 42 participants.
7. Data Display	Table 5 was created to present themes identified in our data along with associated strategies and key findings.
8. Writing Up	Findings are presenting under theme headers with appropriate supporting quotations. We consider the challenges and facilitators associated with each style of coping along with the carers’ change in C-DEMQoL scores and the dyads co-residency status.

## Findings

Five styles of emotion-focused dyadic coping and associated strategies were developed during analysis and are presented in Table 5. These were: (i) common, (ii) supportive, (iii) hostile, (iv) disengaged avoidance, (v) protective (overprotection and protective buffering), reflecting Falconier and Kuhn’s integration model for dyadic coping (2019). Although there were no direct examples of carers saying they asked people with dementia how they were feeling (active engagement), carers mentioned “talking” together (common) and “listening” to the person with dementia (supportive). We therefore considered active engagement to be a component of these supportive dyadic coping styles, rather than a stand-alone theme. We also combined ‘overprotection’ and ‘protective buffering’ into ‘protective,’ as carers used both these styles with the same goal, to shield the person with dementia from distress. We discuss below how each of these dyadic coping styles were used and experienced by carers during the COVID-19 pandemic. Household status and individual changes in carers’ quality of life scores from pre-pandemic to time of interview were also considered.

**Table 5.** Emotion-focused dyadic coping styles and associated strategies.

Coping styles	Coping strategies
Positive	
Common	Team perspective, shared fear, adapting together, shared enjoyment, shared humour, gratitude
Supportive (Active Engagement)	Listening, reassuring, responding appropriately, being flexible, building confidence, hugs, sending gifts
Negative	
Hostile	Shouting, minimising, blaming, questioning, belittling, dismissing, mocking
Disengaged Avoidance	Ignoring, escaping, avoiding conflict, emotional detachment
Protective (Overprotection and Protective Buffering)	Acting, sensitive language, shielding, distracting, hiding emotions

### Common

Many participants, particularly spousal co-residents, approached stress during the pandemic together through communication, shared experiences, and gratitude for one another. These carers referred to the dyad as “a team” and dealt with emotional stress by trying “to talk about everything together”. They used “we” language and positive adjustment and acceptance were common:

I think we just have to accept that that's the way things are at the moment and on the whole we've both accepted it. (Wife B, co-resident)

However, for other participants, attempts to initiate common coping proved distressing or unhelpful. Indeed, some carers who tried to use common coping methods still reported large reductions in quality of life scores during the pandemic. Participants talked about feeling frustrated by failed attempts to cope together when the person with dementia lacked the resources to engage:

Yes, it’s a bit one way, talk to her but I don’t get the right response, ‘I’m listening,’ ‘Yes, yes,’ ‘How are you?’ ‘Fine,’ but how fine is fine I don’t know. (Husband E, co-resident)

One carer talked about being unable to share how she is feeling with her mother because “she [mother] doesn’t want me upset”. This was particularly difficult for the participant during lockdown because she was cut off from other forms of social support and felt like she had “nobody to talk to”. This participant recalled a distressing attempt to engage in common coping as they struggled to find a way to cope together:

I've just burst into tears in front of Mum and said, ‘I can't do this, I can’t cope, I don't know what to do, Mum’ and you know, she's like ‘I don't know what to do either’. (Daughter M, non-co-resident)

There were many carers who said the extra time spent together during COVID-19 lockdown had resulted in a closer relationship with the person with dementia. Shared positive experiences, such as reminiscing, listening to music and sitting in the garden together, meant these dyads enjoyed the time together. One participant highlighted shared humour as a main facilitator in successfully and positively adapting to COVID-19 together as a dyad:

We talk more, we find a lot of humour in a lot of things, we just seem closer somehow, because… I suppose it's because we're thrown together all the time now, and fortunately we've adapted to it. (Wife B, co-resident)

Several carers who discussed common coping described their relationship as full of gratitude and appreciation for one another. Some talked about being “lucky” to have each other, compared to other people who were dealing with the pandemic “alone”.

I think we’re lucky that we’ve got each other, you know, I feel sorry for the people who live alone, because they must suffer… we consider we’re fortunate. (Wife G, co-resident)

### Supportive

Carers discussed different approaches to help the person with dementia manage their emotions. Effective supportive coping involved being sensitive to the persons needs or emotions and responding with empathy and appropriateness. Participants highlighted the importance of “being there” for the person with dementia to “listen to” them and “support as much as you can”. When people with dementia were upset by information about the pandemic, carers talked about needing to provide lots of reassurance:

Sometimes he gets very upset, sometimes he cries about it [COVID-19], you know, ‘Am I going to get that?’ ‘No, you're going to be fine’. (Wife J, co-resident)

One carer mentioned that the pandemic had given him more time to provide supportive coping to his wife. However, this participant appeared to be struggling personally and reported one of the largest reductions in quality of life during the pandemic. He talked about “frustrated” as his family “don’t see what I face” in caring for his wife. When he talked about the ways the pandemic facilitated dyadic coping, he saw the benefits being primarily for the person with dementia:

The pandemic just means we can’t go where we want, but the most basic kind of support for someone is just to be there for them and understand that. So, I mean I think as far not being able to go out as often it's actually helped in that respect in some way. (Husband B, co-resident)

Other participants felt positively about caring for the person with dementia. This was true for carers that had adapted well in the pandemic and had a good relationship. One carer, who reported one of the largest increases in quality of life during the pandemic, talked about feeling appreciated by her husband:

I said ‘What had helped you during this time?’ And he said ‘You’, in other words me, and he added ‘I don't think I'd survive on my own,’ which is quite sweet. (Wife G, co-resident)

Some participants emphasised the importance of tailoring their support according to the needs of the person with dementia. This involved listening to them and thinking about the “most appropriate way of responding at that time”, considering what they needed in the moment was more important than having a specific strategy:

I cannot give you a specific because it varies on a day-to-day what the situation is for me to even calm her down. (Daughter I, non-co-resident)

However, this could sometimes be challenging for carers. Coordinating differences in coping styles could lead to dyadic strain and frustration. While one participant highlighted the importance of tailoring support, he talked about how he could misjudge his supportive coping approach, leading to an increase in negative emotions:

I can’t say I always get it right either. And sometimes I think oh, maybe this is, you know, let's face this anxiety which I think is more head on and that possibly frustrates her sometimes a bit more, a lot, sometimes it doesn’t work right, and sometimes it does work. (Husband A, co-resident)

Some carers also neglected their own coping needs and preferences to accommodate the person with dementia:

We end up doing what makes [WIFE] feel more comfortable anyway, which is fine. She’s the one that has the anxiety. (Husband A, co-resident)

Other participants believed it was important to validate the person with dementia’s emotional experience, whilst also trying to remind them of their strength and abilities to build their confidence:

She’s very down on herself, like ‘I can’t do anything. I don't know what I'm doing. I don’t know why I’m doing it.’ And I said, ‘But, Mum, you’re in the house on your own, you've been... you were married to Dad for 50-odd years, you’ve now dealt… you’re dealing with the bereavement of that, you're still getting up in the mornings, you're still getting dressed, you’re still having a cup of tea, you're still doing what you're doing’. (Daughter M, non-co-resident)

Another common form of supportive coping discussed by participants was physical touch and “hugs”. To protect the person with dementia and adhere to lockdown rules, non-co-resident carers were at times required to socially distance, but physical connection through hugging was so important to carers that many were willing to bend the social distancing rules to hug the person with dementia, even when they felt worried about COVID-19:

I think it was after the last… when the lock-down happened, and Mum was just upset and I went ‘I can't do this anymore, come here, let's have a hug’. (Daughter M, non-co-resident)

### Hostile

Hostile coping stemmed from frustration, blame, and anger towards the person with dementia. Many co-resident carers reported increased interpersonal strain within the dyad during lockdown restrictions. The extra time together led to some dyads feeling “fed up” with each other, meaning participants were less patient and “more snappy”. This negatively impacted dyadic interactions. For co-residents, this was explicit and involved outbursts of emotion directed at the person with dementia:

I will admit it, I do shout at her sometimes. (Grandson A, co-resident)

This often led to the carer feeling remorseful and upset:

As I say, I do get frustrated, and sometimes I lose my cool, which then of course I get all moody and upset with myself for having done that. (Wife E, co-resident)

Other types of hostile coping, mainly used by non-co-resident carers, were more implicit and involved being dismissive of the person with dementia’s emotional experience. This was mainly in evidence where participants believed the person with dementia was not “particularly bothered” by lockdown restrictions due to lack of awareness. For some, even when the person with dementia was clearly voicing negative feelings about the pandemic, the participant still concluded that the emotional impact was low:

I still think she's not upset, but she's aware that she's not seeing family members, she's still aware of that, because she said ‘I hate this virus because I can't see… I can't even see my own family’. (Daughter J, non-co-resident)

Hostile coping was often used by carers that considered the person with dementia’s behaviour to be to some degree intentional, with some accusing them of “telling lies”, or blaming them for their poor emotional state. One participant talked about difficulties with her mother’s coping style, finding it frustrating and difficult to accept:

If she doesn't want to do anything about it her situation won't change. That sounds a bit horrible, I know, but she doesn't... you have to want to do something, don't you, to make it happen, and I think she's just got to the stage now where she just can't be bothered, you know, I won't bother then, I'll just sit here… which is a shame… she keeps saying to me ‘You wait until you’re my age,’ ‘Mum, I would not allow myself to be like this’. (Daughter L, non-co-resident)

Relational tension was also heightened when carers tried questioning the person with dementia about their behaviour, which could lead to an angry response:

He'll look at the same page for, well, for hours on end. I don't know how much of it he's reading, how much of it actually goes in, you know, or whether he's reading the same bit again, or it's just taking him a long time to read it, I don't know, and if I start to question him about things like that, he just gets angry and puts it down. (Wife D, co-resident)

It was sometimes hard to differentiate supportive coping from dismissive hostile coping. Many participants talked about reminding the person with dementia of how “lucky” they were, often comparing their situation to others that might be “worse off” during lockdowns because of ill health, poor living conditions or lack of social support:

I don't let her sit about and feel sorry for herself, it's just not going to achieve anything. I think you've got to be grateful for the things that you've got, and she's been very lucky. (Husband B, co-resident)

While humour could be a positive shared experience between the participant and the person with dementia, it could take the form of mocking. One participant, for example, talked about coping by using the person with dementia as a subject of humour:

You can't explain things to him, I mean we do have a… well, me and him [carer and father] have a laugh, but we probably have a laugh at his [father] expense sometimes, the silly things that he does. (Son D, non-co-resident)

### Disengaged avoidance

Physical and attentional avoidance strategies were used by co-resident carers to avoid dealing with the person with dementia’s emotional state. Many of these participants talked about feeling trapped and overwhelmed during the lockdown; “I can’t get away from him”. Some said they outright “ignore” the person with dementia when it got too much, while others avoided conversation that might encourage an emotional response from the person with dementia:

The worst thing I can say to her is ‘How are you?’ which is the kind of thing… because then that just starts off a whole ‘Oh, woe is me’. (Daughter H, non-co-resident)

Avoidance techniques could be useful in giving the carer and the person with dementia space from each other, particularly during the lockdown. One carer’s strategy to relieve tension and stress was to “hide away for a little bit”:

Leave the room or you know, move... you know, go to the toilet, the toilet's my lifesaver sometimes. (Grandson A, co-resident)

When tensions were high within the dyad, moving away and spending some time apart from each other could be positive, when followed with reconciliation:

He’ll go away and think about it, and come back, and he’s apologetic, he realises he’s over-reacting and over-controlling. (Wife E, co-resident)

Some carers also found it easier to avoid confrontation altogether:

“If he gets a thing in his head, you just agree with him, “Yeah, you’re right, that’s exactly what someone said,” so you try and keep the stress levels down.” (Wife N, co-resident)

Avoidance through emotional detachment was also common for non-co-resident participants. For carers that had never been close to the person with dementia, they concluded that emotional detachment “helps” when caring for the person with dementia. However, those who described a historically close relationship found it “very difficult to break that sort of emotional sort of tie”:

Detach yourself from the loving sort of family you had before… You've got to, you know, the days of, you know, sort of the intimacy and the laughter, the friendship, they seem to go, so you've got to say that… you've got to look at it totally different. It's very hard. (Son A, non-co-resident)

### Protective

All but one carer (Daughter E, non-co-resident) who reported using a protective style of coping also reported a reduction in quality of life during the pandemic (co-resident: Wife A, Wife E, Wife I, Husband C, Husband E; non-co-resident: Sister A, Daughter D). Protective coping involved shielding or distraction techniques used to maintain the emotional state of the person with dementia. For example, carers would go along with the person with dementia to manage their anxiety, such as “pretending to fix something” or avoid triggering language that may elicit an emotional response. Carers also tried to shield people with dementia from negative information surrounding COVID-19. One carer talked about her husband being very “sensitive” to COVID-19 news and would easily become “emotional”. To cope with this, she started directing him away from information that could be distressing:

If it's the news or a report on it, I immediately change the channel he's watching, and find him something that he likes, you know, to distract him. (Wife J, co-resident)

Many participants acknowledged the impact their own emotional state could have on the person with dementia. One participant said she felt stressed about the lack of formal support during the pandemic and was concerned her stress was having a negative impact on her father:

He could feel the stress in me, I'm sure, as well, so that would have had an impact on him, as well. (Daughter O, non-co-resident)

This led to some participants putting their own needs aside to protect the person with dementia’s emotional state. Some participants felt the need to hide their own feelings to protect the person with dementia. For example, one participant was especially worried about his own health problems being exacerbated by long waiting lists due to COVID-19 but felt that he needed to hide his emotions to maintain the emotional wellbeing of his wife:

I thought to myself in this situation you’ve got to be cheerful, if you’re cheerful, [wife]’s cheerful. (Husband E, co-resident)

Another participant talked about struggling to care for her mother during the pandemic. The pressure of trying to hide how she was feeling would eventually become too much and she would “explode” and “verbally lash out” (hostile coping). Despite this, she continued to feel that she needed to hide her emotional state from her mother:

She doesn't want me upset, because I'm her rock. (Daughter M, non-co-resident)

## Discussion

Using data gathered in in-depth qualitative interviews we explored dyadic coping between carers and the people with dementia they care for during the COVID-19 pandemic. Many carers said it was difficult to separate their experiences of coping with the pandemic from coping with their relative’s dementia. This suggests that the dyadic coping styles and dynamics reported here reflect more generally how dyads may cope together with a wide range of commonplace stressors. Participants used a similar range of coping styles and associated strategies to other populations (Falconier & Kuhn, 2019). Carers reported challenges using ‘positive’ styles of dyadic coping, benefits of using some ‘negative’ disengaged avoidance styles, differences in hostile dyadic coping based on co-residency status, and poor outcomes associated with protective dyadic coping. Our findings provide novel contextualised examples of how carers used and experienced dyadic coping styles in the pandemic which may also shed light on these processes at other times.

### Co-residency status

In our sample, the emotions experienced by carers during the pandemic seemed to differ based on their co-residency status. Co-residents talked about spending more time together and, while some felt grateful to be together through the pandemic, others expressed frustration and difficulties finding time away from each other. Alternatively, non-co-residents spent less time together and experienced guilt or disconnect due to social distancing rules. While some worried the person with dementia might feel alone, others felt emotionally detached from the person with dementia. Many non-co-residents also believed the person with dementia wasn’t emotionally impacted by the lockdown restrictions. One support avenue for dyads in the event of a future pandemic could involve training carers and people with dementia to use technology. This could help keep non-co-residents connected and allow co-residents carers to connect with other forms of social support (Hicks et al., 2023).

### Imbalances in positive coping efforts

Carers reported distress with imbalances in positive dyadic coping efforts, in which they were providing all the support and felt they were receiving nothing from the person with dementia. When dyads adopted a successful common approach to coping, they found it easier to adapt to the challenges of COVID-19 together, with some even saying that the extra time together due to lockdowns had improved their relationship. This is consistent with evidence from other populations (Falconier & Kuhn, 2019). However, in our sample, common coping styles could be associated with carer distress when the person with dementia lacked the coping resources to engage or reciprocate. In fact, many carers who preferred common coping methods still talked about feeling alone and frustrated during this lockdown period, and supportive coping was often unreciprocated. This imbalance could leave the carer feeling overwhelmed and neglecting their own individual coping needs by using the person with dementia’s preferred style of coping rather than their own. This is consistent with previous DETERMIND research, suggesting that carers who put the person with dementia’s needs before their own may experience loneliness and reduced quality of life (Hicks et al., 2022; Perach et al., 2022). Interventions that support dyads to establish and maintain a connection to reduce carers’ distress around feelings of coping imbalance should be implemented to support carers’ emotional wellbeing.

### Difficulties using positive coping

Some carers reported increased distress in using positive dyadic coping styles. Providing emotional support to the person with dementia could be challenging and cognitively taxing for carers, requiring them to adapt their coping response to the needs of the person with dementia. It was not enough to simply engage in supportive coping, their attempts needed to be relevant, empathetic and context specific to be effective. This may be particularly difficult for dyads coping with the progressive symptoms of dementia, particularly changes in emotional needs and communication (Henry et al., 2009). Interventions that support people with dementia and carers to identify and share their coping needs may be beneficial. Even when carers did have the skills to provide supportive coping, the emotional burden impacted their own wellbeing. Indeed, previous research has reported an association between emotional empathy and poor mental health outcomes in carers of people with dementia (Hua et al., 2021). Providing people with dementia with alternative sources of emotional support may better sustain the emotional resources carers need to support the person with dementia, as well as to take care of themselves. Although potentially challenging to deliver during a global pandemic, technology could be used to provide dyads with virtual support and connect them to other forms of emotional support (Talbot & Briggs, 2022).

### Avoiding hostile coping

In times of high emotional intensity, carers often used either disengaged avoidance or hostile coping. While these styles are considered maladaptive for non-dementia dyads (Falconier & Kuhn, 2019), avoidance styles were sometimes used to avoid overtly hostile coping, and could be beneficial when followed up with adaptive coping. Consistent with other research (Dixon-Gordon et al., 2015), our data suggests that carers used negative dyadic coping styles when emotional intensity was high. Hostile coping was almost always associated with negative emotions in the carer, such as anger, frustration, and guilt. Conversely, short-term avoidance strategies were often used to protect the carer’s own emotional wellbeing and avoid stressful situations escalating. Dyadic coping interventions that identify precursors and triggers leading to situations of high emotional intensity may give dyads more control in these situations. This should involve an avoidance plan when emotional intensity gets too high, followed by reconciliation training to repair the relationship using adaptive coping strategies. Teaching carers how to identify when their coping resources need replenishing could avoid hostile coping, including emotional or physical abuse within the dyad.

### Explicit or dismissive hostile coping

Some hostile strategies, such as shouting, blaming, and explicit anger towards the person with dementia were mostly used by co-residents. Other more implicit hostile strategies, such as dismissing the person with dementia’s emotional experience, were mostly used by non-co-residents. Dismissive hostile coping - using cognitive reframing strategies on the person with dementia, but without considering their emotional experience - was sometimes hard to distinguish from genuine supportive coping. Many non-co-resident carers who used these strategies believed that the person with dementia had little awareness about the COVID-19 pandemic or felt that its emotional impact on them was low. Conversely, carers who lived with the person with dementia were more likely to report frustration and explicit anger. Interventions should consider the co-residency status of the dyad: those living with the person with dementia may benefit from strategies to manage their emotions, while non-co-resident carers might benefit from connecting and listening to the person with dementia to better understand their emotional experience.

### Maladaptive protective coping

Protective coping may help maintain the emotional state of the dyad short-term, but many carers who reported protective coping also reported reductions in their own quality of life (from pre-pandemic to time of interview). From our data it is unclear whether protective dyadic coping was particularly detrimental to carers’ quality of life during the COVID-19 lockdown, or whether lockdown conditions and caring responsibilities reduced their quality of life and in turn drove their use of protective dyadic coping. Carers reported that distracting or shielding the person with dementia from distressing information, such as COVID-19 news, was associated with less immediate distress. However, carers who used protective coping tended to talk about struggling personally, and some carers believed that hiding their feelings ultimately resulted in a situation in which hostile dyadic coping could occur. It is also useful to note that protective dyadic coping can reduce autonomy (Seaman & Stone, 2017), exacerbating situational difficulties – for example when people with dementia actively employed their own individual coping strategies during the pandemic (Dixon et al., 2022) or if carers misjudge their level of insight (Marzanski, 2000). Using alternative styles of dyadic coping may help dyads maintain their quality of life, particularly when avenues for alternative emotional support are limited. Additionally, support groups and meaningful social connections can offer carers opportunities to vent frustrations and process their emotions openly.

### Strengths and limitations

Our study captured a wide range of lived experiences from carers with varying demographics, dyadic characteristics and reported quality of life scores. We identified factors associated with both effective and less successful emotion-focused dyadic coping attempts. While our findings provide a deep and nuanced picture of how carers utilise and experience dyadic coping, we cannot know which styles of coping are more adaptive in the long-term. Instead, guided by Falconier and Kuhn’s model (2019), we identified challenges associated with each style and have made practical recommendations for intervention. Empirical research is needed to develop and experimentally test our recommendations. Our study focused on the perspective of the carers: to explore these processes more comprehensively additional research is needed from the perspective of people with dementia. Future research could also measure dementia severity and neuropsychiatric symptoms as they may be associated with some of the challenges discussed by carers. Additionally, sampling from a single country (England) may not generalise across all cultures. Finally, due to the pandemic all interviews were carried out remotely and more nuanced information may have been collected with face-to-face interviews.

## Conclusions

Changes in carers quality of life varied during the COVID-19 pandemic, potentially relating to carers co-residency and style of emotion-focused, dyadic coping. Carers discussed a range of experiences and challenges using dyadic coping during lockdowns, highlighting the importance of considering relational, situational, and individual factors that may influence how dyads cope together. Carers reported challenges using ‘positive’ styles of dyadic coping and some benefits using a ‘negative’ (disengaged avoidance) style of coping. Being flexible with dyadic coping, to meet the needs of both members of the dyad, rather than having a specific approach may create better outcomes for carers. This may be particularly beneficial when one member has dementia, as the dyad may need to adapt to changes in both relationship dynamics and the coping resources of the person with dementia. Interventions could support dyads to communicate their coping needs, reconnect following short-term avoidance coping, and identify the type of hostile coping (explicit or implicit) used by carers, which may in turn help tailor support.

## Supplemental Material

Supplemental Material - emotion-focused dyadic coping styles used by family carers of people with dementia during the COVID-19 pandemic Click here for additional data file. Supplemental Material for emotion-focused dyadic coping styles used by family carers of people with dementia during the COVID-19 pandemic by Carmen Colclough, Eleanor Miles, Rotem Perach, Ben Hicks, Josie Dixon, Jennifer Rusted, Kate Gridley, Yvonne Birks, Margaret Dangoor, Paul Donaghy, Riona Mcardle, Elen Moseley, Harsharon K. Sondh, Sube Banerjee in Dementia

Supplemental Material - Emotion-focused dyadic coping styles used by family carers of people with dementia during the COVID-19 pandemic Click here for additional data file. Supplemental Material for Emotion-focused dyadic coping styles used by family carers of people with dementia during the COVID-19 pandemic by Carmen Colclough, Eleanor Miles, Rotem Perach, Ben Hicks, Josie Dixon, Jennifer Rusted, Kate Gridley, Yvonne Birks, Margaret Dangoor, Paul Donaghy, Riona Mcardle, Elen Moseley, Harsharon K. Sondh, Sube Banerjee in Dementia
