# Supplementary material for: Emotion-focused dyadic coping styles used by family carers of people with dementia during the COVID-19 pandemic
Source: Dementia (London). 2023 May 5;22(6):1205–26. doi: 10.1177/14713012231173812 (PMC10164090; doi:10.1177/14713012231173812)
Supplement: Supplemental Material - emotion-focused dyadic coping styles used by family carers of people with dementia during the COVID-19 pandemic [file sj-pdf-1-dem-10.1177_14713012231173812.pdf]

<u>DETERMIND-C19 interview schedule</u>

Purposive sampling criteria:

Aim is to recruit a diverse sample to explore inequalities based on the following sociodemographic determinants:

- Agreed to be interviewed
- Place of residence (rural/urban)
- Ethnicity
- Wealth/income
- Age
- Levels of formal care
- Household size/composition
- Severity of dementia
- Scores on the quantitative components of the research

Researcher note: It is important that you have a good outline of the participant's quantitative responses prior to conducting the interview. The interview will seek to elicit a better understanding of these responses. Make a note of the reasons the participant has been nominated for an interview and reflect on them with the participant and look to delve into them in more detail during the discussion.

Qualitative interview schedule:

The questions are open and broad, and designed so that the participant can lead the interview and discuss those aspects of the pandemic that are pertinent to them. The researcher's role is to prompt on these areas to elicit further insight into the experiences. You do not need to delve into all of the areas and not in the order outlined below. The flow of the interview should be informed by the participant and the areas they want to discuss most.

Remember to encourage the participant to talk in detail about their experiences. Useful prompts will be:

- What makes you say that?
- Can you tell me a bit more about that?
- Can you give me an example of that?

1. **Can you tell me how are things going for you at the moment?**

Potential areas to delve into:

- What is their living situation at the moment and how does this influence their experiences of living with the pandemic?
- What are their daily activities at the moment? Anything helping them to maintain these activities? Any challenges in maintaining their activities?
- What is their relationship like with the carer/person with dementia at the moment?
- How are they being supported? How are they getting supplies such as food/medicines?
- How are they supporting the person with dementia at the moment?

- What is going well?
- What are they finding difficult? What is their health like at the moment? And that of the person with dementia? How are they managing their health conditions?
- Have their/the person's dementia symptoms been affected/changed?
- Are they having much social contact at the moment from family/friends/neighbours? How is this being facilitated and what are the barriers?
- Are they clear about the rules around the easing of the lock-down and how are they finding them? Are there any challenges?
- Are they accessing any dementia services at the moment? If so, how are they finding them?
- What has helped them most during this period?
- What were the biggest challenges for them during this period?

2. **Can you tell me a bit about what things were like for you at the start of the lock-down (March time)?**

Potential areas to delve into:

- How different were they from now? Has anything changed for them?
- How well did they understand the Government restrictions and were they able to follow them? Did they receive a shielding letter?
- How were they finding supporting the person with dementia? What were the challenges? Were they receiving any support?
- How much social contact were they having with family/friends/neighbours? How was this managed?
- Were they making use of any services during this initial period?
- How was their relationship during this period?
- What has helped them to most during this period?
- What were the biggest challenges for them during this period?

3. **How are you feeling about the next few months?**

Potential areas to delve into:

- Do they think much will change in their daily lives?
- Can they foresee any additional challenges?
- What would help them to live better during this period?
- What would help them to better support the person with dementia during this period?

4. **Thinking about everything we have talked about today…**
    a. What is the biggest challenge you think you have faced during this period in relation to supporting someone with dementia?
    b. What has helped you the most in addressing this challenge?
    c. What do you think has been the biggest challenge for the person with dementia
    d. Has anything helped them/you to address this challenge?

e. What other support would you have liked during this time?
f. What advice would you give to other people who have recently been diagnosed with dementia during this pandemic and lock-down?

Other potential questions:

- How are they managing without previously used face-to-face activities/ groups? If they are using online or remote alternatives, how are they finding these?
- Are they finding it easy or hard to find Covid-related information and support they need? If using telephone advice or looking for information online, how are they finding this'?

- Do you feel you are differently placed at the start of the second wave? How?
- What impact have these changes made on your current circumstances?
- Possibly ask Carer how well the PWD understands the situation and the concept of "Pandemic" and how they feel they would have responded pre dementia? How has this affected the carer?

5. Is there anything else you would like to discuss that we have not touched on?
