# Supplementary material for: Emotion-focused dyadic coping styles used by family carers of people with dementia during the COVID-19 pandemic
Source: Dementia (London). 2023 May 5;22(6):1205–26. doi: 10.1177/14713012231173812 (PMC10164090; doi:10.1177/14713012231173812)
Supplement: Supplemental Material - Emotion-focused dyadic coping styles used by family carers of people with dementia during the COVID-19 pandemic [file sj-pdf-2-dem-10.1177_14713012231173812.pdf]

Supplementary 2.

Examples of coping strategies coded for each style of coping

<u>Coping Styles</u><br>Coping Strategies	Family carer quote
<u>Common</u>	
Team perspective	"'We try to talk about everything together, yeah, just try and be a team on it." *(Wife E, co-resident)*
Shared fear	"We are terrified of catching it, terrified" *(Wife N, co-resident)*
Adapting together	"I suppose it's because we're thrown together all the time now, and fortunately we've adapted to it. She is aware that other people have found it difficult but 'it's worked all right with us." *(Wife B, co-resident)*
Shared enjoyment	"Just sit out there and listen to the birds singing, and watch the trees blowing. We like that, we like that, simple life." *(Wife N, co-resident)*
Shared humour	"'We talk more, we find a lot of humour in a lot of things, we just seem closer somehow." *(Wife B, co-resident)*
Gratitude	"I think we're lucky that we've got each other." *(Wife G, co-resident)*
<u>Supportive</u>	
Listening	"It's just listen to them [people with dementia] and just be a support, support as much as you can." *(Sister A, non-co-resident)*
Reassuring	"Sometimes he gets very upset, sometimes he cries about it, you know, 'Am I going to get that?' [COVID-19], 'No, you're going to be fine'." *(Wife J, co-resident)*
Responding appropriately	"I tend to try to think about what is the most appropriate way of responding at that time, rather than having a thought out 'This is how I'm going to respond every day that that happens'." *(Husband A, co-resident)*
Being flexible	"We end up doing what makes [wife] feel more comfortable anyway, which is fine. She's the one that has the anxiety level." *(Husband A, co-resident)*
Building confidence	"It's just building Mum back up again, to say you're good at things, you can do these all right, you are okay." *(Daughter M, non-co-resident)*
Hugs	"The physical side of giving Mum a hug, yes, I do it now, because Mum needs them." *(Daughter M, non-co-resident)*
Sending gifts	"I tend to go every couple of days, just to drop something off, to let her know that I'm thinking about her." *(Daughter B, non-co-resident)*
<u>Hostile</u>	
Shouting	"I will admit it, I do shout at her sometimes." *(Grandson A, co-resident)*
Minimising	"I still think she's not upset, but she's aware that she's not seeing family members,

	she's still aware of that, because she said 'I hate this virus because I can't see... I can't even see my own family'." *(Daughter J, non-co-resident)*
Blaming	"You had all the equipment to do it and you still fell because you didn't use it." *(Daughter A, non-co-resident)*
Questioning	"He's reading the same bit again, or it's just taking him a long time to read it, I don't know, and if I start to question him about things like that, he just gets angry and puts it down." *(Wife D, co-resident)*
Belittling	"She keeps saying to me 'You wait until your my age', 'Mum, I would not allow myself to be like this, you know'." *(Daughter L, non-co-resident)*
Dismissing	"I don't let her sit about and feel sorry for herself, it's just not going to achieve anything. I think you've got to be grateful for the things that you've got, and she's been very lucky, very lucky. There's people who are in hospital, who've had strokes." *(Husband B, co-resident)*
Mocking	"We probably have a laugh at his expense sometimes, the silly things that he does." *(Son D, non-co-resident)*
<u>Disengaged Avoidance</u>	
Ignoring	"She'll be moaning on and on and on, you can ignore it you know." *(Grandson A, co-resident)*
Escaping	"Leave the room or you know, move... you know, go to the toilet, the toilet's my lifesaver sometimes." *(Grandson A, co-resident)*
Avoiding conflict	"It's trying just to move around sort of not get irritated with each other, sort of walk away… if he gets a thing in his head, you just agree with him, 'Yeah, you're right, that's exactly what someone said', so you try and keep the stress levels down" *(Wife N, co-resident)*
Emotional detachment	"So, it's very difficult to break that sort of emotional sort of tie, but you've got to change it, you've got to change your attitude." *(Son A, non-co-resident)*
<u>Protective</u>	
Acting	"[I] try and fix her computer all the time, not that I know how to do it, but she's always whinging that it's not working, and shouting and that, you've got to pretend you're fixing something." *(Daughter E, non-co-resident)*
Sensitive language	"Mum doesn't really know she's got dementia and if the word gets mentioned, she's not entirely comfortable with that, so I just talk about her memory loss." *(Daughter D, non-co-resident)*
Shielding	"If it's the news or a report on it [COVID-19], I immediately change the channel he's watching." *(Wife J, co-resident)*
Distracting	"I could read things in the paper that interest him, just in that moment, or talk about something, you know, that's the only thing I can do, take his mind off it." *(Wife I, co-resident)*
Hiding emotions	"She doesn't want me upset, because I'm her rock." *(Daughter M, non-co-resident*
